# Supplementary material for: Association between oral microbiome and five types of respiratory infections: a two-sample Mendelian randomization study in east Asian population
Source: Front Microbiol. 2024 Apr 10;15:1392473. doi: 10.3389/fmicb.2024.1392473 (PMC11039966; doi:10.3389/fmicb.2024.1392473)
Supplement: Supplementary file 1 [file Data_Sheet_1.zip › Supplementary Materials/MR plots of saliva/Chronic sinusitis/s__Prevotella_conceptionensis_mgs_303/forest.pdf]

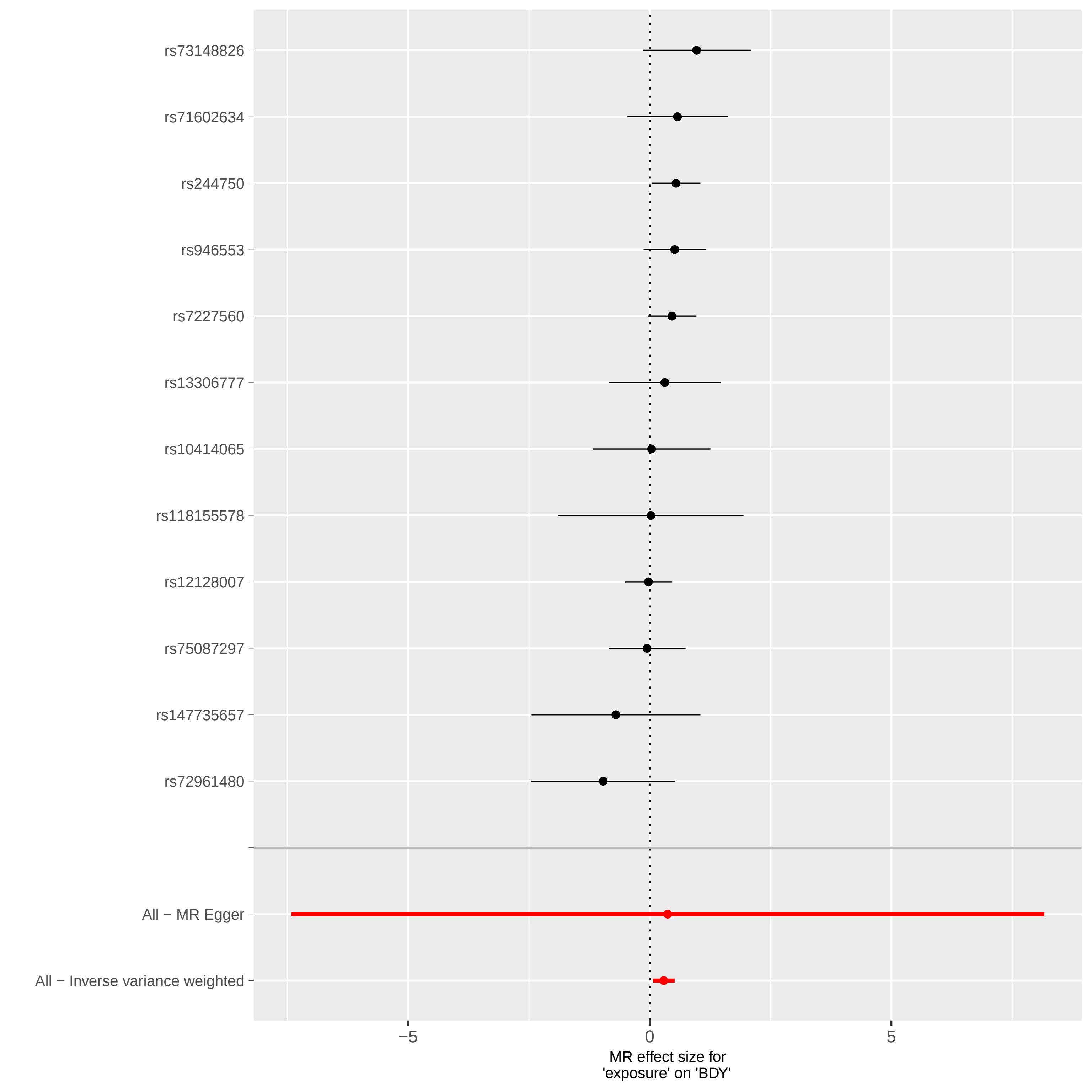

rs73148826
rs71602634
rs244750
rs946553
rs7227560
rs13306777
rs10414065
rs118155578
rs12128007
rs75087297
rs147735657
rs72961480
All − MR Egger
All − Inverse variance weighted
−5
0
5
MR effect size for
'exposure' on 'BDY'
